# Primary Sjögren’s Syndrome Presenting in Adolescence: A Case Report

**DOI:** 10.7759/cureus.97074

**Published:** 2025-11-17

**Authors:** Sutlan Albaqawi, Ahmed Alanazi, Ruqayyah H Almarshadi

**Affiliations:** 1 Oral and Maxillofacial Surgery, King Khalid Hospital, Hail, SAU

**Keywords:** dry eye and mouth, oral and maxillofacial pathology, primary sjogren’s syndrome, salivary gland diseases, sjogren's disease

## Abstract

Early-onset primary Sjögren’s syndrome is rare and may present atypically in pediatric patients. We report a 14-year-old female who presented with bilateral parotid swelling and oral dryness. Contrast-enhanced computed tomography revealed diffuse glandular enlargement with numerous tiny punctate radiopaque foci. Minor salivary gland biopsy confirmed focal lymphocytic sialadenitis with approximately 50 lymphocytes per high-power field and mild acinar atrophy. Diagnosing this syndrome in children can be challenging due to its rarity and overlapping symptoms with other inflammatory or infectious conditions. Recognizing these subtle early features is essential, as delayed diagnosis may lead to irreversible glandular damage and systemic complications. This case emphasize the importance of early recognition and supports the diagnostic value of minor salivary gland biopsy in confirming the disease.

## Introduction

Sjögren’s syndrome (SS) is a systemic autoimmune exocrinopathy in which epithelial-immune interactions drive lymphocytic infiltration of the salivary and lacrimal glands, leading to mucosal dryness and a spectrum of systemic manifestations [1,2]. The condition predominantly affects women and incidence increases with age, although presentation in younger patients, including adolescents, is increasingly recognized and can be diagnostically challenging [2,3].

Current classification relies on the 2016 American College of Rheumatology/European League Against Rheumatism (ACR/EULAR) criteria, which assign weight to anti-Sjögren's-syndrome-related antigen A (anti-SSA (Ro)) serology and objective glandular tests. However, seronegative phenotypes are recognized, necessitating tissue-based confirmation in selected cases [4]. Beyond histopathology, salivary gland ultrasound (SGUS) has emerged as a valuable non-invasive tool to complement biopsy for both diagnosis and follow-up [5]. Despite advances, clinical heterogeneity is frequent, extraglandular involvement is common, and no universally effective disease-modifying therapy has been established [6].

Importantly, SS confers an elevated risk of B-cell lymphoma, reinforcing the need for vigilant assessment when new focal salivary lesions or systemic “red flags” arise [7]. Against this backdrop, we report a 14-year-old female patient with atypical features of SS presenting to the emergency department, with diagnosis confirmed by lower-lip minor salivary gland biopsy, illustrating the value of early, multimodal evaluation in non-classic, pediatric presentations.

## Case presentation

A 14-year-old female patient presented to the emergency department with bilateral parotid swelling and oral dryness of gradual onset. There was no history of fever, suppuration, trauma, or medication use associated with xerostomia. On examination, both parotid regions were mildly enlarged and non-tender, without palpable nodules or lymphadenopathy. Intraoral examination revealed reduced salivary pooling and dry mucosa, with otherwise normal dentition and gingiva.

**Figure 1:**
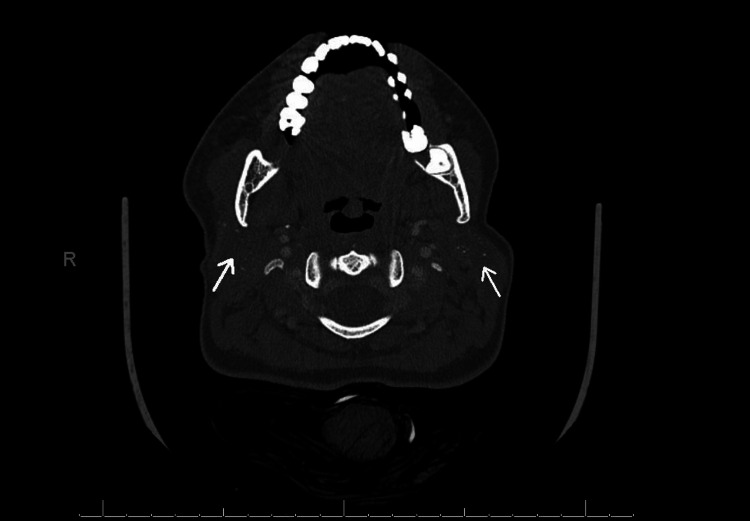
CECT of the parotid glands showing diffuse glandular enlargement with tiny punctuate radiopaque foci scattered bilaterally (arrows), suggestive of chronic sialadenitis consistent with early-stage Sjögren’s syndrome CECT: Contrast-enhanced computed tomography

Given the bilateral glandular enlargement, contrast-enhanced computed tomography (CECT) of the head and neck was performed, revealing diffuse enlargement of both the parotid glands with numerous small, punctate radiopaque dots scattered throughout the glandular parenchyma (Figure 1).

No focal mass, abscess, or ductal obstruction was identified, consistent with chronic inflammatory sialadenitis.

**Figure 2:**
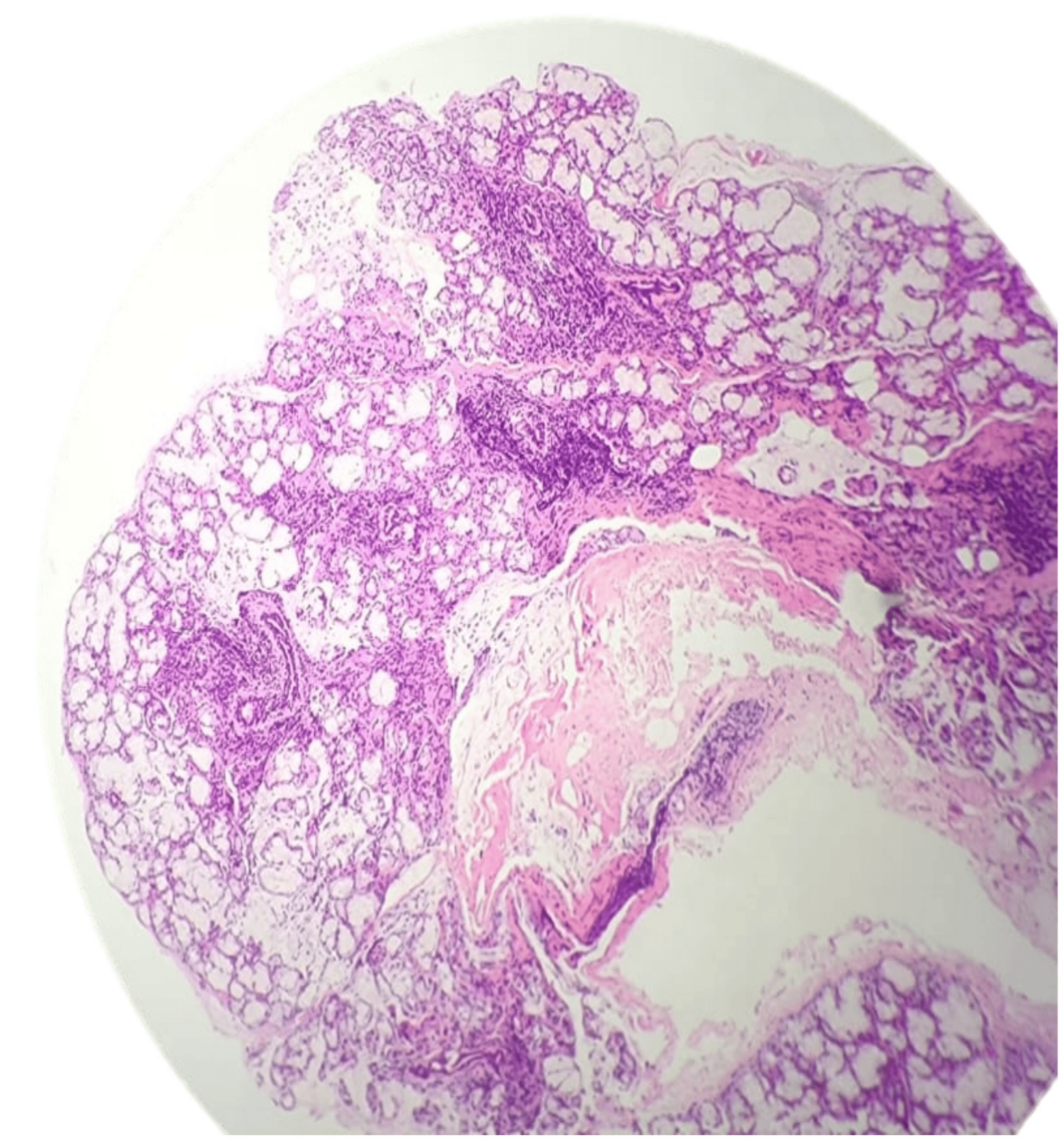
Section of salivary gland lobules and acini showing focal nodular lymphatic aggregate >50 lymphocytes in a periductal distribution

A lower-lip minor salivary gland biopsy was performed, demonstrating focal lymphocytic sialadenitis with approximately 50 lymphocytes per high-power field in a periductal distribution, mild acinar atrophy, and no germinal center formation (Figure 2). 

According to the Chisholm and Mason classification, this corresponds to Grade 3-4, or a focus score ≥1, which is considered diagnostic for primary SS [8]. This finding confirmed the diagnosis in this early-onset case, consistent with previous reports of pediatric SS [3-9]. The patient was referred for rheumatologic assessment and symptomatic management, including saliva substitutes and ophthalmologic follow-up.

## Discussion

Primary SS is a chronic autoimmune disorder, characterized by lymphocytic infiltration of exocrine glands, leading to xerostomia, keratoconjunctivitis sicca, and systemic manifestations [1]. While SS typically affects middle-aged women, it can rarely present in children and adolescents. Juvenile or early-onset cases are often underdiagnosed due to atypical presentation. Studies report pediatric SS presenting between three and 14 years of age, often with salivary gland enlargement and oral dryness as initial manifestations [2-9]. Our patient, a 14-year-old girl, highlights this unusual early-onset presentation, emphasizing the importance of considering SS even in adolescents.

Imaging plays a supportive role in evaluating salivary gland involvement. SGUS has gained prominence for diagnosis and follow-up [5]. However, in emergency settings or when ultrasound is not available, CECT can provide a detailed evaluation of glandular architecture and exclude obstructive or neoplastic causes. In this case, CECT demonstrated diffuse enlargement with tiny punctate radiopaque foci in both parotid glands, consistent with chronic inflammatory sialadenitis and suggestive of autoimmune involvement.

The diagnosis of SS is challenging, particularly in seronegative or early-onset cases. Minor salivary gland biopsy remains the gold standard, confirming the presence of focal lymphocytic sialadenitis [4]. Histopathology in our patient revealed periductal lymphocytic aggregates (~50 lymphocytes per high-power field) with mild acinar atrophy, consistent with focal lymphocytic sialadenitis. According to the Chisholm and Mason classification, this corresponds to Grade 3-4, or a focus score ≥1, which is considered diagnostic for SS [8]. The biopsy provides definitive confirmation, particularly in young or seronegative patients, and guides early management.

SS demonstrates significant clinical heterogeneity, including potential extraglandular involvement [6]. Management remains largely symptomatic, including saliva substitutes, cholinergic agents, and monitoring for ocular complications. Importantly, SS carries an increased risk of B-cell lymphoma, reinforcing the need for vigilant follow-up [7]. This case highlights the unusual presentation of SS in an adolescent, the supportive role of CECT, and the diagnostic reliability of minor salivary gland biopsy.

## Conclusions

SS, though uncommon in children, should be considered in cases of unexplained salivary gland swelling. In this young patient, CT imaging and minor salivary gland biopsy provided complementary evidence that supported an early diagnosis. This case reinforces the importance of multimodal evaluation and tissue confirmation when assessing atypical, early-onset presentations of this autoimmune disorder. While this report emphasizes the value of early recognition, it remains limited as a single case and cannot represent the full spectrum of pediatric presentations. Larger pediatric studies are needed to better define early diagnostic indicators and guide age-specific evaluation strategies. Ultimately, early identification is crucial, as timely management may help prevent disease progression and improve long-term outcomes.
